# What Is New in the Treatment of Smoldering Multiple Myeloma?

**DOI:** 10.3390/jcm10030421

**Published:** 2021-01-22

**Authors:** Niccolo’ Bolli, Nicola Sgherza, Paola Curci, Rita Rizzi, Vanda Strafella, Mario Delia, Vito Pier Gagliardi, Antonino Neri, Luca Baldini, Francesco Albano, Pellegrino Musto

**Affiliations:** 1 Division of Hematology, Fondazione IRCCS Ca’ Granda Ospedale Maggiore Policlinico, 20122 Milano, Italy; niccolo.bolli@unimi.it (N.B.); antonino.neri@unimi.it (A.N.); luca.baldini@unimi.it (L.B.); 2 Department of Oncology and Onco-Hematology, University of Milan, 20122 Milan, Italy; 3 Unit of Hematology and Stem Cell Transplantation, AOUC Policlinico, 70124 Bari, Italy; nicolasgherza@libero.it (N.S.); paolacurci@tiscali.it (P.C.); rita.rizzi@uniba.it (R.R.); mario.delia74@gmail.com (M.D.); vitopier86@gmail.com (V.P.G.); francesco.albano@uniba.it (F.A.); 4 Department of Emergency and Organ Transplantation, “Aldo Moro” University School of Medicine, 70124 Bari, Italy; vandastrafella@gmail.com

**Keywords:** smoldering multiple myeloma, immunotherapy, clinical trials, progressive disease, prognostic factors, quality of life, overall survival

## Abstract

Smoldering multiple myeloma (SMM), an asymptomatic plasma cell neoplasm, is currently diagnosed according to the updated IMWG criteria, which reflect an intermediate tumor mass between monoclonal gammopathy of undetermined significance (MGUS) and active MM. However, SMM is a heterogeneous entity and individual case may go from an “MGUS-like” behavior to “early MM” with rapid transformation into symptomatic disease. This wide range of clinical outcomes poses challenges for prognostication and management of individual patients. However, initial studies showed a benefit in terms of progression or even survival for early treatment of high-risk SMM patients. While outside of clinical trials the conventional approach to SMM generally remains that of close observation, these studies raised the question of whether early treatment should be offered in high-risk patients, prompting evaluation of several different therapeutic approaches with different goals. While delay of progression to MM with a non-toxic treatment is clearly achievable by early treatment, a convincing survival benefit still needs to be proven by independent studies. Furthermore, if SMM is to be considered less biologically complex than MM, early treatment may offer the chance of cure that is currently not within reach of any active MM treatment. In this paper, we present updated results of completed or ongoing clinical trials in SMM treatment, highlighting areas of uncertainty and critical issues that will need to be addressed in the near future before the “watch and wait” paradigm in SMM is abandoned in favor of early treatment.

## 1. Introduction

In the continuous spectrum of monoclonal gammopathies, smoldering multiple myeloma (SMM) occupies an intermediate position between what is usually referred to as a pre-malignant condition, i.e., monoclonal gammopathy of indeterminate significance (MGUS) and active multiple myeloma. SMM has a higher disease burden than MGUS, but does not show end-organ damage or any of the other myeloma-defining events observed in MM [1,2,3,4,5]. SMM is, therefore, regarded to as an asymptomatic cancer.

SMM is rare, representing 8–14% of the total of MM patients. The median age of onset is 67 years. The incidence is 0.4 cases per 100,000 people per year and is higher in Americans of African descent, as reported for MM [6,7]. The median time to progression (TTP) of SMM to active MM is about 5 years. However, this is not constant: it approximates 10% per year during the first 5 years, then decreases to 3% per year for the subsequent 5 years and slows significantly after 10 years, reaching a progression rate of 1% similar to what reported for MGUS [7,8]. SMM is therefore heterogeneous and cases cover an extremely wide spectrum. Some are biologically “MGUS-like”, i.e., indolent conditions with a higher tumor burden and in fact one third of SMM patients will never progress in their life. At the other end of the spectrum are “de facto MM” cases, i.e., patients with a biologically aggressive disease, just not meeting clinical criteria at the time of analysis. Such a variety of clinical behaviors represents a challenge for prognostication and management of individual patients [9].

The conventional approach to SMM is classically represented by watch and wait to monitor possible disease progression, delaying treatment to that time. In a way, clinicians would request laboratory and imaging tests to decide “when” to treat SMM. However, advances in the knowledge of disease biology have led to improved SMM risk stratification. Furthermore, novel and effective treatments produce much-improved survival in MM patients. Therefore, it is time for re-consider the current approach and ask whether we are ready to decide “which” SMM patient to treat instead of “when” [10]. Early treatment could indeed delay progression to MM or avoid it altogether, potentially curing some patients and prevent the severe complications of end-organ damage. Not surprisingly, early treatment is a much-debated issue [4,11,12,13,14,15].

## 2. Pathogenesis and Diagnosis of Smoldering Multiple Myeloma

From a biological point of view, SMM shows the initiating events of all monoclonal gammopathies, i.e., recurrent translocations of oncogenes to the immunoglobulin heavy chain (IGH) locus on chromosome 14, or multiple trisomies of odd-numbered chromosomes [16]. However, these events are required but not sufficient for progression to active MM. Indeed, progression for SMM to MM is characterized by an ongoing acquisition of additional genomic events, each of which confers a distinct risk. While the genome of ultra-high risk SMM shows a landscape of mutations and chromosomal abnormalities that is more similar to MM [17], abnormalities like del (13q), amp (1q), del (17p) increase in frequency as the disease shifts to active MM [18]. Among other genomic events associated with progression are translocations between the IGH locus and the MYC oncogene [17,19] and accumulation of complex rearrangements [17,20]. Last, the activity of several mutational processes can be tracked over time. Initiating lesions arise from the activity of the DNA activation-induced cytidine deaminase (AID) within the germinal center, while late mutations associated with progression to MM arise from the aberrant activity of the APOBEC (apolipoprotein B mRNA editing catalytic polypeptide-like) family of cytidine deaminases, a mutational process common to many cancers and absent in normal plasma cells (PCs) [17,21,22].

Genomics is however not part of routine SMM diagnosis. For this purpose, International Myeloma Working Group (IMWG) criteria are used, taking into account several disease burden measures. Patients with SMM have more than 3 gr/dL of monoclonal component and/or >10% clonal bone marrow plasma cells (BMPC). In addition, SMM has no evidence of end-organ damage, amyloidosis and “myeloma defining events”, i.e., >60% BMPC, a free lights chain (FLC) ratio >100 and >1 focal lesion with magnetic resonance imaging (MRI) [3]. IMWG recommends SMM is monitored at 2–3 months following initial diagnosis, then every 4–6 months for one year and every 6–12 months after that if stable [23]. However, follow-up intervals also depend on the individual risk of progression, which can be measured by several different approaches

## 3. Risk Stratification of Smoldering Multiple Myeloma

In the last few decades, several risk scores for SMM progression have been proposed. Some include laboratory parameters that are routinely used in the clinic such as the BMPC infiltration rate, immunoparesis of non-involved immunoglobulins, serum MC concentration, an altered FLC ratio and albumin levels [8,24,25]. Others also include more sophisticated multi-parametric flow-cytometry assessment of BMPCs or clonal plasma cells circulating in peripheral blood [26,27,28]. Prognosis has however been found to correlate with a variety of factors. Examples include the increase over time of MC and Bence–Jones proteinuria [29,30], the decrease of hemoglobin [31], specific features of the BM biopsy [32] or bone involvement detected by MRI or PET-CT [33,34]. Of particular interest are prognostic factors that take into account the intrinsic features of the biopsy, particularly the cytogenetic and molecular findings of the clonal population [35,36,37,38], or their gene expression profiling [39].

However, many of these studies have lost much of their value since the 2014 IMWG revised SMM diagnostic criteria [3]. Since then in fact, several cases that would have been classified as high-risk smoldering are now considered as active MM and treated as such. Furthermore, some discordances among different SMM risk models have been reported [40]. This requires more modern prognostic scores that incorporate the 2014 diagnostic criteria and are universally applicable. The recent “20/2/20 SMM score” was inspired by this need and developed based on three simple criteria: involved to uninvolved serum FLC ratio > 20, M-protein > 2 g/dL and BMPC > 20%. The absence of these criteria, or the presence of 1 or 2+, stratifies patients in three risk groups with 109.8, 67.8 and 29.2 months median time to progression in low, intermediate and high-risk groups, respectively [41]. Since biological features of the disease are indeed causal to its clinical behavior, the inclusion of cytogenetic data to this prognostic score further refines its accuracy. In particular, assessment by FISH of t (4;14), t (14;16), del (13q) and amp (1q), together with a risk score built on the same FLC ratio, BMPC and M-protein concentration as above returns a four-group risk stratification model. Two year progression rates are 72.5%, 51.1%, 26.2% and 3.8% for the high, intermediate, intermediate-low and low-risk groups, respectively [42].

Although times are not ready yet for a routine application of genomic techniques for SMM prognostication [43], clear differences emerge from NGS analysis of indolent vs. progressive asymptomatic plasma cell dyscrasias [17,44,45,46]. A “genomic model” of SMM risk of progression has been proposed that includes mutations in *TP53* and *ATM*, del (17p), *KRAS* and *NRAS* gene mutations, *MYC* aberrations (translocations or copy-number variations) [45]. The absence of these alterations conferred a median time to progression of 7.2 years, while patients with one or more progressed in a median time of 1.2 years and faster if two or more alterations were present.

## 4. Initial Studies on Early Treatment of Smoldering Multiple Myeloma

The concept of early treatment in SMM can be applied with different intensities and goals. Treatment can be low-intensity to delay progression, similar to MM, or even more intense with the goal to reach a cure (Table 1).

Initial approaches to early treatment in SMM have not been successful, but outcomes have improved dramatically since the advent of novel treatments. A recent meta-analysis evaluated eight randomized, controlled trials for a total of 885 patients where early vs. deferred treatment in SMM was compared [64]. The studies ranged from “old” approaches as melphalan plus prednisone [65,66,67] or bisphosphonates +/− thalidomide [68,69,70], to treatment with novel agents, specifically siltuximab [62] or lenalidomide and dexamethasone [47,48]. It was found that early treatment significantly decreased the progression of SMM with a risk ratio (RR) of 0.53 (95% CI 0.33–0.87, *p* = 0.01). The benefit was more significant in patients receiving melphalan plus prednisone or immunomodulatory drugs. Furthermore, when stratified by risk according to the Spanish criteria [26], the high-risk subgroup showed the most benefit in terms of reduction of progression, (RR = 0.51, 95% CI 0.37–0.70, *p* = 0.0001), as importantly showed an improvement in overall survival (OS) as well (RR = 0.53, 95% CI 0.29–0.96, *p* = 0.04). Regarding adverse events, early treatment significantly increased the risk of secondary primary malignancies (SPM) (RR = 4.13, 95% CI 1.07–15.97, *p* = 0.04). Thalidomide and lenalidomide specifically caused constipation. Infections were not affected by early treatment.

## 5. Lenalidomide-Based Treatments for Smoldering Myeloma

The most mature and convincing data on early treatment of SMM with novel agents derive from randomized phase-3 prospective studies on using lenalidomide with or without dexamethasone. The studies intended to provide a mild and tolerable treatment aimed at delaying SMM progression.

The pivotal QuiRedex trial (NCT00480363) enrolled 119 patients with high-risk SMM. This was defined by the presence of >10% BMPC and M-protein > 3 g/dL. In the presence of only one feature, patients were required to have >95% aberrant BMPC by immunophenotyping and immunoparesis. The treatment consisted in nine 4-week induction cycles with lenalidomide plus dexamethasone and lenalidomide maintenance for 2 years. The control group underwent observation [47,48]. The latest update at a median follow-up of 10.8 years showed an RR of 0.54 for death (95% CI, 0.3 to 0.9; *p* = 0.034) and 0.27 for progression (95% CI, 0.16 to 0.42; *p* < 0.0001) in the early treatment group as compared to observation [49]. The median time to progression (TTP) was 9.0 vs. 2.1 years and median OS was not reached yet in the treatment arm vs. 7.8 years in the control arm. This OS benefit was confirmed whether high-risk SMM was defined by the Mayo or by the Spanish criteria. More SPM were observed in the treatment group (10% vs. 2% in the control group), although the cumulative risk showed only a trend towards significance (*p* = 0.07). While treatment was likely successful thanks to its anti-myeloma activity, immunophenotypic studies suggested that lenalidomide also enhanced the immune surveillance against the tumor switching from a tolerogenic to an effector microenvironment in high-risk SMM patients [71]. One important caveat in these studies concerns the risk of the early treatment impacting the efficacy of the subsequent anti-myeloma treatment at the time of progression. However, no differences were observed in OS between groups when analyzed from the start of the anti-MM treatment at progression. This suggests that early treatment with lenalidomide does not induce chemoresistant clones and early therapy in SMM phase does not negatively impact on the following treatments.

The positive effects of lenalidomide on progression and survival of SMM patients were confirmed in a subsequent study. In the ECOG E3A06 phase 3 trial (NCT01169337) lenalidomide single-agent was compared with observation in patients with intermediate or high-risk SMM [50]. Treatment was offered until SMM progression, toxicity, or withdrawal for other reasons. The primary endpoint was progression-free survival (PFS), where progression was defined on either clinical or biochemical bases. Expectedly, lenalidomide reduced the tumor burden in 50% of patients vs. 0% in the control arm. Importantly, after a median follow-up of 35 months, PFS was significantly longer in the lenalidomide arm than in the observational arm, with an RR of 0.28 (95% CI, 0.12 to 0.62; *p* = 0.002). The benefit was indeed striking, resulting in a 72% reduction in the risk of progression to symptomatic disease. This reduction was seen across all patients, but was most relevant in the high-risk SMM group, which included, however only 25 patients. In this study, SMM risk was defined by Mayo Clinic criteria, intermediate risk patients were included and the significance was retained using either 2008 [24] or 2018 [41] definitions. Data on OS are not available yet and of the six deaths in the study, two occurred in the lenalidomide group and four in the control group. Hematological toxicities were as expected in the treatment arm, where non-hematological grade 3 or 4 adverse events also occurred in 28% of patients. Notably, in this US trial, discontinuation rate was quite high (50%, 40% of which due to adverse events). SPMs were seen in 5.2% vs. 3.5% of treated vs. untreated patients, respectively.

## 6. Proteasome Inhibitor-Based Treatments for Smoldering Myeloma

The concept that SMM is an early stage plasma cell neoplasm, without some of the complexity of active MM [72], makes it tempting to think that a more intense treatment approach may result in cure of some SMM cases rather than just progression delay, a goal not achievable when treatment is started at the active MM stage [73]. Consequently, several more intensive treatments are being tested in SMM. Such approaches generally include triplets with a proteasome inhibitor (PI), an immunomodulatory drug (IMID) and dexamethasone. In some instances, treatments include monoclonal antibodies or even autologous stem cell transplantation (AuSCT). Results of such approaches are less advanced than those of lenalidomide-based combinations. Most are pilot studies with a limited number of patients and the few phase 2 trials are very recent and results are still preliminary. However, these are approaches worth describing below.

Eighteen high-risk SMM patients were treated with the potent carfilzomib-lenalidomide-dexamethasone (KRd) regimen in the US pilot study NCT01572480, resulting in an overall response rate (ORR) of 100% [51,74]. Importantly, minimal-residual disease (MRD) negativity, assessed by multiparametric flow cytometry (MFC) (92%) or NGS (75%), was observed at very high rates. The median potential follow-up was 43.3 months. At the analysis cutoff date, 63% of patients remained MRD-negative and the estimated 4-year PFS and OS were 71% and 100%, respectively. Reported toxicities were in line with previous experience with this combination. A subsequent phase 2 study from the same group included 52 patients with high-risk SMM diagnosed according to Mayo Clinic or PETHEMA risk scores. Treatment consisted of eight cycles of KRd followed by lenalidomide maintenance for 24 cycles (KRd-R). The median potential follow-up was 27.3 months. All patients responded to treatment and 78% of patients achieved stringent CR. The primary endpoint was MRD-negative CR rate, which was 70.2%, with a median duration of 5.5 years. Results were therefore very favorable. Indeed, only 10% of patients developed MM at the 5-year landmark analysis. No deaths were reported. Grade 3–4 treatment-related AE occurred in 33% of patients and included cytopenias, thromboembolism, skin rash and lung infections [52].

A multi-center, open-label phase 2 trial promoted by HOVON group is ongoing. Here, high-risk SMM patients defined according to either the Mayo or Spanish criteria and patients with ultra-high-risk SMM as defined by IMWG, are randomized with KRd vs. Rd, followed by lenalidomide maintenance for two years (NCT03673826). Results are still awaited and will provide a direct comparison of the efficacy of the regimens described above.

Some studies explored an even more intense approach to SMM with the intent to cure the disease. The phase 2 GEM-CESAR is a single-arm trial, focusing on high-risk and ultra-high risk SMM patients [53,54]. Six 4-week cycles of KRd were administered as induction, followed by high-dose melphalan and AuSCT, consolidation with two KRd cycles and finally by lenalidomide-dexamethasone maintenance for up to 2 years. Authors set a primary endpoint consisting of sustained MRD negativity of at least 50% measured by next-generation flow (NGF). At the latest update [54] ORR was 98% post-induction, 98% post-AuSCT and 100% post-consolidation; 68.6% of patients reached ≥ CR post-consolidation, with 55% of them achieving flow MRD negativity.

The all-oral triplet ixazomib-lenalidomide-dexamethasone (IxRd) is being tested in a phase 2 trial for patients with high-risk SMM [75], where a nine-cycle induction schedule is followed by 15 cycle of maintenance with ixazomib and lenalidomide. Based on preliminary results in the first 26 patients, ORR was 89% with a CR rate of 19.2% and no progression to overt MM at the time of the analysis. Toxicities were mild and manageable. An even milder regimen of ixazomib and dexamethasone has been tested in a pilot study based on 14 patients with high-risk SMM [55] (NCT02697383). At a median follow-up of 17 months, 10 patients were on treatment, nine of which reached at least VGPR. No patient had progressed to symptomatic MM. Three patients experienced grade 3 non-hematological adverse events, including two lower respiratory tract infections and one intestinal complication.

## 7. Monoclonal Antibody-Based Treatments for Smoldering Myeloma

Similar to more advanced stages of the disease, monoclonal antibodies are also being tested in SMM [76]. The anti-CD38 monoclonal antibody daratumumab is evaluated in three treatment schedules (extended intense, extended intermediate, or short dosing) in the randomized phase 2 CENTAURUS study (NCT02316106) [56]. The study enrolled 123 patients with intermediate/high-risk SMM. Analysis performed at a median follow-up of 26 months showed CR rates of 4.9%, 9.8% and 0% in the intense, intermediate and short dosing cohorts, respectively. In the same cohorts, two-year PFS rates were 89.9%, 82% and 75.3%, respectively. Progressive disease (PD)/death rate per patient-year, defined as the number of patients who progressed to MM or died divided by the total duration of progression-free survival (PFS) for all patients, in patient-years, was significantly favorable in all arms and consistent with a projective median PFS > 24 months. Interestingly, the authors observed that target-saturating trough concentrations were preserved in the intense dosing arm, but less so in the intermediate and or short dosing schedules, potentially contributing to the efficacy differences. No new safety issues occurred.

These data prompted a subsequent randomized phase 3 study based on the long dosing schedule, called AQUILA (NCT03301220), where daratumumab is compared to watch and wait [77]. This study is based on a flat-dose, subcutaneous administration of daratumumab at 1.800 mg. Doses are weekly for cycles 1 and 2, every 2 weeks for cycles 3–6 and every 4 weeks thereafter, for up to 39 cycles. The study will include approximately 360 high-risk SMM patients. High-risk will be defined as the presence of at least one of the following: serum M-protein ≥ 3 g/dL, IgA isotype, reduction of two uninvolved immunoglobulin levels, serum FLC ratio ≥ 8 < 100, BMPC > 50% < 60%.

Daratumumab efficacy will also be tested in combination with lenalidomide, following in vitro demonstration of synergy between the two molecules [78] and excellent data in relapsed-refractory MM setting [79]. The ongoing DETER-SMM trial (NCT03937635) is a phase 3, randomized trial comparing lenalidomide plus dexamethasone vs. the triple combination of daratumumab, lenalidomide and dexamethasone, with OS as a primary endpoint. The study aims at an enrollment of 280 patients with high-risk SMM diagnosed up to one year earlier and having the following high-risk features: abnormal serum FLC ratio (≤0.125 or ≥8.0) with the involved chain measuring < 100 mg/L and/or serum M-protein > 3 g/dL and/or presence of any the adverse cytogenetic events t (4;14), del (17p), 1q gain. This study again confirms the varied approach to the definition of high-risk SMM and the need for a consensus for stratification in future trials.

In an even more intense schedule, the ongoing phase 2 ASCENT trial (NCT03289299) proposes the quadruple combination of daratumumab, carfilzomib, lenalidomide and dexamethasone (DKRd) without AuSCT in subjects with high-risk SMM. Patients will receive a total of 12 DKRd cycles, followed by maintenance with lenalidomide and daratumumab for 12 cycles. Despite the study being still ongoing, preliminary safety data have been recently reported for 46 patients. Grade 3–4 adverse events consisted in cytopenias, thromboembolic events, infections, hypertension, diarrhea and allergic reactions. Each occurred in less than 10% of patients, but 52% experienced at least one grade > 2 adverse events. Up to 17% of patient required a dose modification, mostly for carfilzomib, but the relative median dose intensity for the drugs were 85%, 92%, 80% and 98% for carfilzomib, daratumumab, lenalidomide and dexamethasone, respectively, across the delivered cycles, demonstrating the feasibility of such an approach [57].

A complementary question concerns the utility of pharmacological intervention in lower risk SMM cases. Daratumumab is being evaluated in such setting in a single-arm, phase 2 study (NCT03236428). The pre-specified primary endpoint is a VGPR or better after 20 cycles. The schedule consists in single agent i.v. infusions every week for cycles 1–2, every two weeks for cycles 3–6 and every four weeks for cycles 7–20 [80]. Of 28 patients enrolled, 15 completed at least 6 cycles. No patient progressed to MM, ORR was 53% and at least VGPR was achieved in 20% of patients. Treatment was well tolerated, with no deaths or therapy discontinuation.

Isatuximab is an anti-CD38 monoclonal antibody that targets a different epitope than daratumumab. It is currently trialed in a phase 2 study (NCT02960555) in high-risk SMM, defined according to the PETHEMA criteria. Treatment is administered as four weekly infusions (20 mg/kg iv) at cycle 1, every two weeks at cycles 2–6 and every four weeks at cycles 7–30. The primary endpoint is ORR [58]. Twenty-four patients were evaluated with a median number of cycles administered of 11.5 (range 6–30). Treatment was stopped in 5 patients, of which two because of progression to active MM after 6 and 16 months. No deaths have been reported but five grade 3 treatment-related adverse events occurred. These consisted of dyspnea as part of infusion-related reactions, headache, neutropenia, urinary tract infections and all resolved to baseline. ORR was 64% with 10 PR, 4 VGPR, 1 CR with MRD negativity by MFC at 10^−5^. Importantly, health-related quality of life (HRQoL) scores were reported in this study. Isatuximab may reduce anxiety and worries of progression to MM and the score improved after 6 cycles.

Isatuximab will also be evaluated in combination with lenalidomide and dexamethasone in a phase 3, randomized multicenter study, where the control arm will consist of lenalidomide and dexamethasone (NCT04270409). The study population will include 300 patients within 5 years of high-risk SMM diagnosis per IMWG criteria. The study will be preceded by a safety run-in phase to confirm the recommended dose of isatuximab within the proposed combination. Primary endpoints will be safety and efficacy, the latter measured as PFS. Secondary endpoints will be represented by pharmacokinetic and immunological studies, ORR, duration and quality of responses (including MRD), type of progression, time to second progression, OS, economic and HRQoL evaluations.

Elotuzumab is an anti-SLAMF7/CS1 monoclonal antibody. The drug is thought to work mostly through antibody-dependent cellular cytotoxicity (ADCC) mediated by CD56 NK cells and monocytes/macrophages [81]. The NCT01441973 phase 2 trial enrolled patients with SMM to receive two different schedules of elotuzumab [59]. ORR was 10%, 2-year PFS 69% and grade 1–2 infections of the upper respiratory tract and IRRs were 58% and 13%, respectively. Despite its proposed mechanism of action, no relationship emerged between baseline CD56dim NK cells and response. Overall, results of the trial confirmed the lack of significant activity of elotuzumab single-agent already seen in symptomatic MM.

Elotuzumab has also been evaluated in combination with lenalidomide and dexamethasone (EloRd) in patients with high-risk SMM in another phase 2 trial (NCT02279394) [60]. EloRD induced PR or better in 41 out of 50 patients, including three CR and 18 VGPR. No progression to overt symptomatic MM was observed and the toxicity profile was manageable.

Pembrolizumab is an anti PD-1 monoclonal antibody, a check-point inhibitor with in vitro immunologic activities in asymptomatic multiple myeloma [82]. The drug was investigated in 13 patients with intermediate/high-risk SMM (NCT02603887) [61]. After a median of eight cycles, most patients (85%) had a stable disease. Interestingly, while one patient progressed, another carrying adverse high-risk features (17p deletion and a high-risk gene-expression signature) reached a sustained MRD negativity. Three patients discontinued the treatment due to immune-related adverse events.

Interleukin 6 (IL-6) is a cytokine that favors neoplastic plasma cell growth. The IL-6 blocking antibody siltuximab was tested in a randomized, double-blind, placebo-controlled study that enrolled 85 patients with high-risk SMM (NCT01484275) [62]. The study’s primary end-point was 1 year PFS, which resulted in 84.5% in the experimental arm compared to 74.4% in the placebo arm at a median follow-up of 29.2 months. Median PFS with siltuximab was not reached, while it was 23.5 months in the placebo arm (*p* = 0.057). Adverse events in the experimental arm were mainly of grade 2/3. Infections and urinary complications were common and some severe. Three deaths occurred with siltuximab and four with placebo.

## 8. Other Therapeutic Approaches for Smoldering Myeloma

For decades a strategy aimed at stimulating the patient’s own immunological response against the clonal BMPCs has been envisioned. After the failure of patient-specific anti-idiotype vaccines, a more modern approach is the use of PVX-410, a vaccine carrying a combination of peptides from selected over-expressed antigen targets on clonal PCs, i.e., XBP1, CD138 and CS1/SLAMF7. This vaccine is employed in a multicenter, dose-escalation phase 1/2a study (NCT01718899). A total of 22 intermediate/high-risk SMM patients received PVX-410, with or without lenalidomide [63]. The vaccine resulted in mild-to-moderate injection site reactions and constitutional symptoms in a subset of patients and proved to be immunogenic as demonstrated by an increase in frequencies of IFN-γ positive effector T lymphocytes specific for the PVX-410 tetramer. Comparing the two arms of the study, the addition of lenalidomide correlated with even higher frequencies of such lymphocytes and of vaccine-specific effector memory cells, which were also more persistent. In terms of efficacy, three of 12 patients progressed in the PVX-410-alone cohort, with a median TTP of 36 weeks. Conversely, in the combination cohort 5 of 12 patients showed a clinical response, one patient progressed and the median TTP was not reached. This promising vaccine is also under investigation combined with the selective histone-deacetylase inhibitor citarinostat +/− lenalidomide in a phase 1 clinical trial (NCT02886065) in the SMM setting. Again, in the vaccine field, a study is investigating a PD-L1 peptide vaccine as a way to elicit an immune response against this pathway in SMM (NCT03850522).

Not all studies showed promising efficacy in SMM. A phase 2 trial with Ibrutinib (NCT02943473), a Bruton tyrosine kinase inhibitor which acts in the downstream proliferation and activation pathways of B-lymphocytes, was recently closed due to poor accrual and unfavorable risk/benefit ratio in patients with high-risk SMM, suggesting that this pathway is not relevant for clonal plasma cell survival.

## 9. Conclusions

Of all the trials described, only one has so far reported a significant OS benefit for early treatment in high-risk SMM [48]. While it is likely that, in due course, others will follow, the effect has already been achieved to refuel enthusiasm in the field of early treatment in these patients. Though exciting, results should also be interpreted with caution due to the small sample sizes and improved diagnostic techniques and criteria in recent years. Heath-related quality of life of SMM patients receiving potentially undue therapy is another important issue that should be taken into account [12,83].

However, looking at the studies that are currently ongoing, all seem to consider improvement in survival as a foregone finding in the context of high-risk SMM treatment, since the control arm is much more frequently represented by a lenalidomide-dexamethasone than placebo. This concept seems to be supported by genetic studies, showing how high-risk SMM is indistinguishable from active MM [17,45]. The logical consequence of this observation though should be that these high-risk cases deserve an intense treatment as much as active MM cases do. Low-intensity approaches aimed at delaying progression were probably justified 10 years ago, when first envisioned, but seem to be less so now.

Overall, we think that patients with lower risk SMM should not receive any treatment; in these subjects only active observation should be therefore pursued. Regarding, instead, high-risk SMM, a great debate is still present in the hematological community. We argue that patients presenting (or progressively developing) the presence of multiple clinical and biological risk factors (i.e., significant increase of serum MC, FLC ratio and BMPC infiltration, decrease in hemoglobin levels, high-risk cytogenetics) might benefit from an early treatment. If so, it seems to day appropriate to hypothesize that these patients should be not differently treated from those with active MM; these therapies, however, should be still performed within the context of clinical trials, whose final results about OS and HRQoL are eagerly awaited. Though validated data are still not available, one could also hypothesize that, in these patients still without end-organ damage, treatments might be better tolerated than in those with advanced MM. In these cases, however, patients should be always adequately informed about risk/benefit ratio of such an approach, and also in terms of HRQoL, considering that no treatment, even those less aggressive with lenalidomide +/− dexamethasone, though supported by prospective, randomized trials, has been so far approved for patients with SMM.

Another relevant observation is that risk stratification in SMM needs to be urgently standardized. Current, randomized studies have discordant criteria for risk stratification, hampering the future possibility of applying their conclusions to current risk definitions [42] right from the start. Furthermore, now that NGS has proved to be applicable to clinical-grade MM diagnostics [43,84,85], the question is also whether the increased amount of information that comes with it will be useful for SMM risk stratification, perhaps even through non-invasive techniques [86,87]. In the meantime, the current diagnostic criteria and treatment landscape mandates that the diagnostic approach to asymptomatic gammopathies is carried out with great care to identify patients with active MM or high-risk SMM, even if treatment is not offered at the asymptomatic stage. In this setting, aside from standard blood and urine laboratory tests, FISH or validated equivalent molecular techniques on purified BMPCs should be considered to implement risk stratification. Particularly, the recently developed, IMWG endorsed prognostic score includes variables that reflect both disease burden and biological features of SMM and can be measured by most centers [42]. At present time, it seems therefore an acceptable, feasible compromise for risk-adapted monitoring of SMM patients in the daily practice and their selection in future clinical trials.

Diagnostic imaging is equally important and should consist of a low-dose whole-body CT scan (LDWBCT) and whole-body MRI if LDWBCT is negative. Axial MRI or whole-body PET-CT are reasonable alternatives [34]. Overall, longitudinal, risk-adapted, monitoring is crucial in SMM, since changes in laboratory or imaging studies are the most sensitive markers of progressive disease requiring careful evaluation for starting a therapeutic approach.

In conclusion, the promised advantage of early treatment in SMM and the technological progress mandate that eventually a consensus is reached on what clinical, laboratory, genomic and imaging features are actually needed to identify truly aggressive cases at the asymptomatic stage. Furthermore, in a possible future biology-driven classification, a question also arises as to whether aggressive cases should be treated like active MM, rather than less intensively.

## Figures and Tables

**Table 1:** Selected clinical trials in smoldering multiple myeloma.

Reference	Clinical Trial	Phase	N. Patients	Treatments	Follow-Up	Response Rates	Outcomes
[47,48,49]	QuiRedex NCT00480363	III	119	**Induction:** 28 day-cycle (C1–9) Lenalidomide 25 mg p.o. days 1–21 + Dex 20 mg p.o. days 1–4, 12–15; **Maintenance:** 28 day-cycle (C1–24) Lenalidomide 10 mg p.o. days 1–21 (N. 57) **Observation** (N. 62)	Median FU: 10.8 years	After induction: ORR: 79%, CR: 14% After maintenance: ORR: 90%, CR: 26% ORR/CR: NA	Median TTP: 9.0 vs. 2.1 years (*p* = 0.034) Median OS: NR vs. 7.8 years (*p* < 0.0001)
[50]	SWOG E3A06 NCT01169337	III	182	**Continuous therapy:** 28 day-cycle (C1–PD): Lenalidomide 25 mg p.o. days 1–21 (*n* = 90) **Observation** (*n* = 92)	Median FU: 35 months	ORR: 50%, CR 0%	3-year PFS: 91% vs. 66% (*p* = 0.002)


						ORR: NA	
[51,52]	NCT01572480	II	18	**Induction:** 28 day-cycle (C1–8): Carfilzomib 20/36 mg/sqm i.v. days 1,2, 8, 9, 15, 16 + Lenalidomide 25 mg p.o. days 1–21 + Dex 20 mg (C1–4) and 10 (C5–8) p.o. or i.v. days 1, 2, 8, 9, 15, 16 **Maintenance:** 28 day-cycle (C1–24) Lenalidomide 25 mg days 1–21	Median FU: 43.3 months	ORR: 100%, MRD negativity: 92% by MFC, 75% by NGS	Estimated 4-year PFS: 71% Estimated 4-year OS: 100%
[53,54]	GEM-CESAR NCT02415413 67	II	90	**Induction:** 28 day-cycle [C1–6]: Carfilzomib 20/36 mg/sqm i.v. days 1,2, 8, 9, 15, 16 + Lenalidomide 25 mg p.o. days 1–21 + Dex 40 mg days 1, 8, 15, 22 **AuSCT:** Melphalan 200 mg/sqm **Consolidation:** 2 cycles as induction **Maintenance:** 28 day-cycle Lenalidomide 10 mg days 1–21 + Dex 20 mg days 1, 8,15, 22 for 2 years	Median FU: 32 months	ORR: 98% post-induction, 98% post-AuSCT, 100% post-consolidation and maintenance; ≥CR: 38.4%, 61.5% and 68.6% at the same time-points MRD negativity: 23%, 44% and 55% at the same time points	PFS 93% (5 biochemical progressions)
[55]	NCT02916771	II	26 (56 planned)	**Induction:** 28 day-cycle (C1–9): Ixazomib 4 mg p.o. days 1, 8, 15 + Lenalidomide 25 mg p.o. days 1–21 + Dex 40 mg p.o. days 1, 8, 15, 22 **Maintenance:** 28 day-cycle (C10–24): Ixazomib 4 mg p.o. days 1, 8, 15 + lenalidomide 15 mg p.o. days 1–21 (*n* = 45)	Median number of cycles: 8	ORR: 89%, CR: 19.2%	No progression to MM
[56]	CENTAURUS NCT02316106	II	123	Dara 16 mg/kg IV in 8-wk cycles: **Extended intense** (N. 41): (C1) every 1 week; (C2–3) every other week; (C4–7) every 4 week; (C8-20) every 8 week **Intermediate intense** (N. 41): (C1) every 1 week and (C2–20) every 8 week **Short dosing** (N. 41): (C1) every 1 week	Median FU 26 months	ORR:56%, CR: 4.9%, ORR:54%, CR: 9.8%, ORR:38%, CR: 0%	2-year PFS: 89.9% vs. 82.0% vs. 75.3%
[57]	ASCENT NCT03289299	II	46 (83 planned)	**Induction:** Carfilzomib (36 mg/sqm twice weekly i.v. or as per updated protocol 56 mg/sqm i.v. weekly for 2 weeks), Lenalidomide (25 mg daily p.o. for three weeks), Dara (weekly for 8 doses i.v., every other week for 16 weeks), Dex 40 mg weekly p.o., in 4 week cycles for 6 cycles. **Consolidation:** the same previous regimen with Dara i.v. every 4 weeks and Dex 20 mg weekly p.o. for another 6 cycles. **Maintenance:** 12 cycles with Lenalidomide (10 mg daily p.o. for three weeks) and Dara i.v. (day 1 every other cycle) of a 4-week cycle.	NA	NA	Only preliminary safety data available: Single grade 3–4 adverse events < 10%; 52% at least one grade > 2 adverse events; relative median dose intensity: 85% (carfilzomib), 92% (dara), 80% (lenalidomide) and 98% (dexa).
[58]	NCT02960555	II	24 (61 planned)	**Isatuximab 20 mg/kg i.v.** in 4 week cycle (C1) every 1 week; [C2–6] every other week; [C7–30] every 4 week	Median number of cycles: 11.5	ORR: 64%, CR: 5%, with MRD negativity	NA
[59]	NT01441973	II	31	**Elotuzumab 20 mg/kg i.v.** [C1] days 1, 8, then (C2-progressive disease) monthly every 4 weeks **Elotuzumab 10 mg/kg i.v.** [C1] days 1, 8, 15, 22, then (C2-progressive disease) monthly every 2 weeks	FU at least 28 months	ORR: 10% (cumulative)	2-year PFS 69% (cumulative)
[60]	NCT02279394	II	50	**Induction:** 28 day-cycle (C1–2) Elotuzumab 10 mg/kg i.v. days 1, 8, 15, 22 + Lenalidomide 25 mg p.o. days 1–21 + Dex 40 mg p.o. days 1, 8, 15, 22 (C3–8): Stem cell collection; Elotuzumab 10 mg/kg i.v. days 1, 15 + Lenalidomide as 25 mg p.o. days 1–21 + Dex 40 mg p.o. days 1, 8, 15 **Maintenance:** 28 day-cycle (C9–24) Elotuzumab 10 mg/kg i.v. days 1 + Lenalidomide 25 mg p.o. days 1–21	NA	ORR: 84%, CR: 6%	No progression to MM
[61]	NCT02603887	Pilot study	13	Pembrolizumab 200 mg IV every 3 weeks × 8 cycles; with option to continue up to 24 cycles if continued benefit	Median number of cycles: 8	ORR: 8%, CR: 8%, MRD negativity 8%	15% of patients progressed to MM
[62]	NCT01484275	Pilot study	85	**Siltuximab 15 mg/kg i.v.** in 2 h q 4 week (N. 43) (C1–progressive disease) (N.43) **Observation** (N. 42)	Median FU 29.2 months	NA	1-year PFS: 84.5% vs. 74.4% (*p* < 0.06) Median PFS: NR vs: 23.5 months; OS NR in both arms
[63]	NCT01718899	I-IIa	20	**PVX-410 vaccine cohort** (*n* = 3 + 6) 0.4-0.8 mg SC; every 2 weeks × 6 doses **PVX-410 combination cohort** (*n* = 10): PVX-410 vaccine 0.8 mg SC every 2 weeks × 6 doses + Lenalidomide 25 mg p.o. D1-21 every 28 days × 3 cycles	NA	Immune response: 95%; (10/11 PVX-410 monotherapy, 9/9 PVX-410 combination)	PVX-410-alone: 3 progressions to MM (median TTP 36 weeks) Combination cohort: 1 progression to MM, median TTP NR

Abbreviations: AuSCT: autologous stem cell transplantation; C: cycle; CR: complete response; Dara: daratumumab; Dex: dexamethasone; FU: follow-up; i.v.: intravenous; MRD: minimal residual disease; NA, not available; NGF: next generation flow; NGS: next generation sequencing; NR: not reached; ORR: overall response rate; OS: overall survival; PD: progressive disease; PFS: progression-free survival; p.o.: per os; PR: partial response; s.c.: subcutaneous; TTP: time-to-progression; VGPR: very good partial response.

## Data Availability

All data presented derive from published papers.
